# Current Epidemiological and Clinical Features of Sarcoidosis in Japan: A Nationwide Claims Data Study

**DOI:** 10.7759/cureus.83328

**Published:** 2025-05-01

**Authors:** Koichi Miyashita, Keita Hashimoto, Shotaro Maeda, Takafumi Suda

**Affiliations:** 1 Second Division, Department of Internal Medicine, Hamamatsu University School of Medicine, Shizuoka, JPN; 2 Medical Affairs, Kyorin Pharmaceutical Co. Ltd., Tokyo, JPN

**Keywords:** claims data, corticosteroids, cumulative incidence, epidemiology, prevalence, sarcoidosis

## Abstract

Background: Comprehensive epidemiological and geoepidemiological data on sarcoidosis in Japan, particularly including mild cases, remains limited. This study investigated the prevalence, cumulative incidence, and clinical characteristics of sarcoidosis using large-scale claims data.

Methods: This retrospective study analyzed data from the Deidentified Scrubbed Claims Healthcare Database (DeSC), covering approximately 20 million individuals in Japan. We investigated patients diagnosed with sarcoidosis between April 2014 and October 2023. The prevalence and cumulative incidence were calculated for fiscal year 2021 (FY2021), and patient characteristics, including comorbidities and treatments, were analyzed.

Results: In FY2021, the estimated prevalence and cumulative incidence of sarcoidosis were 73 and 9.0 per 100,000, respectively. Among 10,910 cases analyzed for clinical characteristics, 68.8% were female individuals, with a mean age of 69.7 years for female individuals and 62.6 years for male individuals. No clear regional differences were observed in prevalence or cumulative incidence (not tested). Oral corticosteroids were prescribed to 25.2% of patients, with a median annual dose of 1,645 mg. Other systemic treatments were prescribed to <3% of patients. The common comorbidities included type 2 diabetes mellitus (21.6-50.0%), sleep disorders (25.1-36.0%), and fractures (8.2%). Sarcoidosis was also associated with other autoimmune diseases, including rheumatoid arthritis, Sjögren syndrome, and autoimmune thyroiditis.

Conclusion: The results of this study provide new insights into sarcoidosis epidemiology and clinical characteristics in Japan, including treatment patterns and comorbidities. These findings suggest potential shared immunological mechanisms among various autoimmune diseases and highlight the need for comprehensive management approaches that consider both disease-specific and treatment-related complications.

This article was previously presented as a meeting abstract at the 65th Annual Meeting of The Japan Respiratory Society on April 12, 2025.

## Introduction

Sarcoidosis is an inflammatory disease of unknown etiology characterized by non-caseating granulomas in multiple organs [1,2]. Its pathogenesis likely involves an immune response triggered by an antigen and underlying genetic predisposition. Infectious antigens, including *Mycobacterium tuberculosis* and *Cutibacterium acnes*, may also be associated with this condition [1,3]. While sarcoidosis can affect various organs including the skin, eyes, heart, and nervous system, it most commonly involves the lungs and lymph nodes [3,4]. Symptoms vary based on the affected site: patients with lung involvement often experience respiratory symptoms such as cough and shortness of breath, whereas other patients experience systemic symptoms such as fever, fatigue, and malaise [5,6]. Although spontaneous remission is common, sarcoidosis may also become chronic or refractory, leading to decreased patient quality of life (QOL). Treatment is primarily pharmacotherapy, most commonly corticosteroids and immunosuppressive agents [7,8].

Sarcoidosis occurs worldwide, with varying prevalence among countries and regions. Recent epidemiological studies using national medical databases have reported prevalence and incidence rates per 100,000 persons, respectively, of 59 and 7.6 in the United States [9], 106 and 18 in Finland [10], 143 and 6.8 in Canada [11], and 160 and 11.5 in Sweden [12], although these reports utilize different databases, leading to variations in patient demographics, sarcoidosis severity, and access to healthcare. In contrast, nationwide epidemiological studies of sarcoidosis are limited in Japan. A previous study of 1,027 patients with pathologically confirmed sarcoidosis reported an incidence rate of 1.01 per 100,000 people [13]. Although a large-scale analysis was also conducted using clinical survey records [14], the survey primarily included patients with severe diseases requiring treatment. Consequently, no nationwide epidemiological data, particularly on mild cases and asymptomatic cases under observation, have been reported.

Medical databases contain data on sarcoidosis cases, including mild cases, and the use of claims data allows for an epidemiological analysis that includes these patients. Therefore, the aim of the study was to investigate the incidence and prevalence of sarcoidosis in Japan, along with patient demographics including sex, age, comorbidities and medication status, using a large-scale claims data base.

## Materials and methods

Data source and study population

This retrospective descriptive study analyzed data from the Deidentified Scrubbed Claims Healthcare Database (DeSC), a Japanese claims database, to investigate the epidemiology, diagnosis, and treatment of sarcoidosis in Japan. The database includes data from the National Health Insurance, Health Insurance Association, and Advanced Elderly Medical Service System and comprises data for approximately 20 million individuals who existed between April 2014 and October 2023. This study enrolled patients diagnosed with sarcoidosis (International Classification of Diseases 10th Revision (ICD-10) code D86 (sarcoidosis)) [15]. In addition, glycated hemoglobin (HbA1c) data were collected from health check-up records to evaluate blood glucose status only for patients for whom data were available.

**Figure 1:**
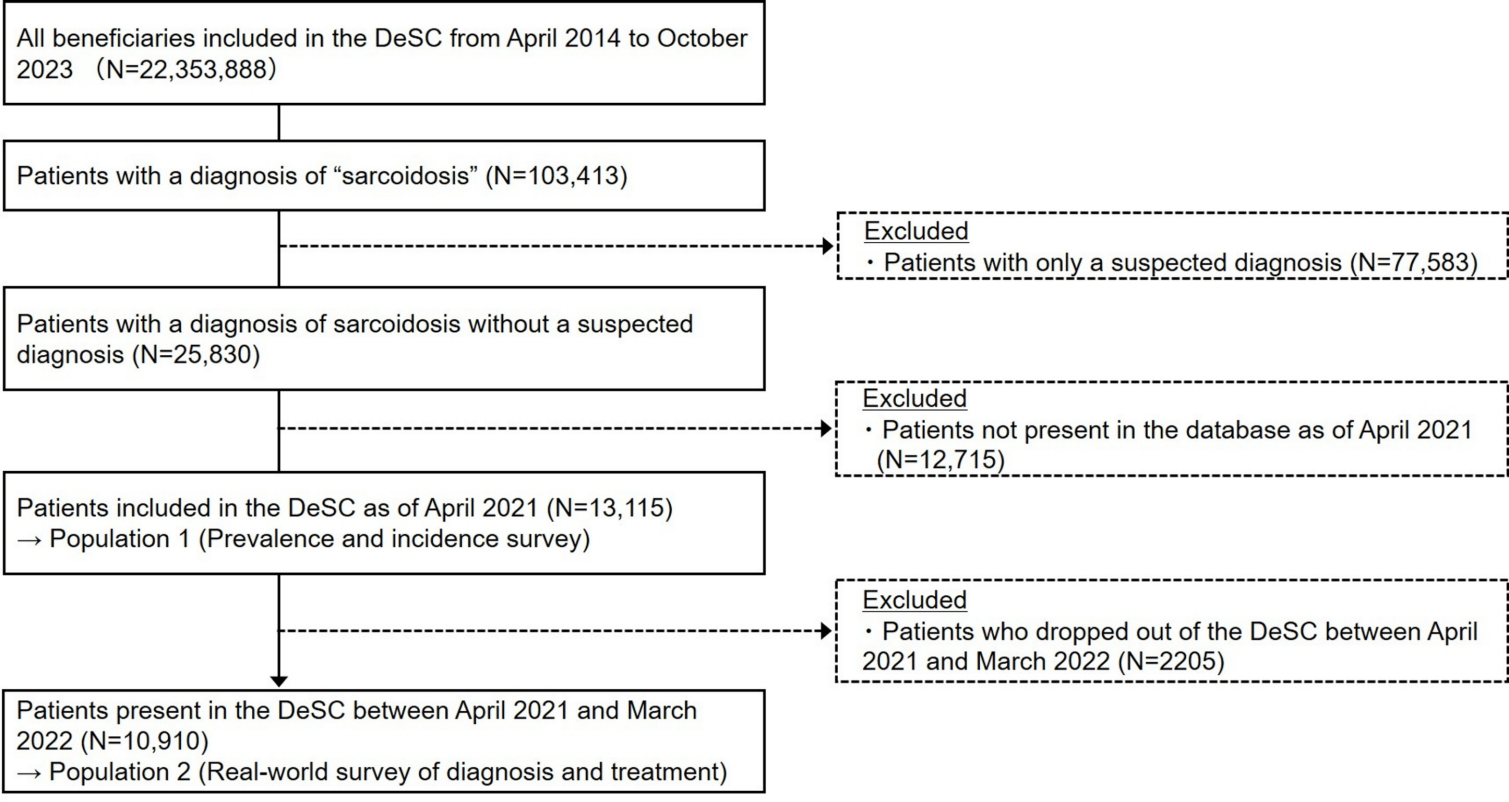
Flow of patient deposition DeSC, Deidentified Scrubbed Claims Healthcare Database

To calculate the prevalence and cumulative incidence for the fiscal year 2021 (FY2021, April 2021 to March 2022), we extracted data on patients included in this database as of April 2021, diagnosed with sarcoidosis during FY2021, and without suspected diagnoses (Population 1). For the real-world survey of diagnosis and treatment, to prevent underestimation due to differences in the observation period, we selected patients who did not drop out of the data during FY2021 (Population 2). The patient deposition flow is shown in Figure 1.

Study variables

The variables included in this descriptive study were age, sex, locations (payer and medical institute), payment of specific medical expenses according to the “Act on Medical Care for Patients with Intractable Diseases” [16], sarcoidosis type, comorbidities, and treatments. Health check-up data were also collected when available.

The sarcoidosis types were classified according to the lesion sites, as described in the disease names from the claims data. Data for the lesions in the lungs, heart, skin, and eyes were summarized. Previous epidemiological studies reported the lung as the most common sarcoidosis lesion site (86.0-95.5%) [4,13]. However, in Japan, more than half of all cases of sarcoidosis are recorded simply as “sarcoidosis” with no indication of the lesion location, making it the most common type. Therefore, since pulmonary sarcoidosis is often referred to simply as "sarcoidosis" without specifying the organ, unlike other organs where the lesion site is typically mentioned, only the lung was explicitly defined as a disease entity. This definition was based on clinical experience, considering commonly co-occurring conditions. Cases diagnosed as sarcoidosis lacking a description of the lesion location were categorized as “pulmonary sarcoidosis” if bilateral hilar lymphadenopathy (BHL), diffuse interstitial pneumonia, or pulmonary fibrosis were also described on the same claim (Table 1).

**Table 1:** Definitions of pulmonary sarcoidosis according to the combinations of disease names The analysis excluded idiopathic conditions (e.g., idiopathic pulmonary fibrosis), focusing exclusively on non-idiopathic etiologies

Disease name		
Pulmonary sarcoidosis		
Sarcoidosis	and	Hilar lymphadenopath
		Diffuse pulmonary fibrosis
		Pulmonary fibrosis
		Diffuse interstitial pneumonia
		Interstitial pneumonia

Regarding comorbidities, we defined diseases reportedly comorbid with sarcoidosis and associated with steroid use based on ICD-10 codes and disease names. In addition, to improve the validity of the diagnosis, we also tabulated disease names/codes that are combined with therapeutic treatments commonly used for each disease (see Appendix A). Sarcoidosis treatments were tabulated using the Anatomical Therapeutic Chemical Classification System (ATC) codes [17] listed in the Japanese Sarcoidosis Treatment Guidelines [18]. The annual number of oral corticosteroid (OCS) prescriptions was calculated by converting prednisolone equivalents to previously reported potency values [19].

Statistical analyses

We determined the prevalence and incidence rates using the DeSC database and calculated the expansion factors for each age and sex group using national census data reported by the Statistics Bureau of the Ministry of Internal Affairs and Communications. These were then applied to the data of patients with sarcoidosis to calculate the estimated number of patients [20]. The present study included patients diagnosed with sarcoidosis during FY2021, with no prior diagnosis before April 2021. The prevalence and cumulative incidence rates in each region were calculated by dividing the number of cases in each region by the number of individuals in the DeSC. Because regional data were not available from the Health Insurance Association, only data from the National Health Insurance and Advanced Elderly Medical Service System were collected. However, when grouped by region, there are several age groups with extremely small sample sizes, leading to overestimation. For this reason, we used actual measured values based on data for regional prevalence and incidence rates, rather than estimates.

For descriptive statistics, means (SD) or medians (IQR) were calculated for continuous variables, whereas frequencies and proportions (%) were calculated for categorical variables. Because this was a descriptive study, no statistical tests were conducted. R version 4.4.0 was used for all statistical analyses [21]. The results of the study are reported in accordance with the recommendations of “REporting of studies Conducted using Observational Routinely-collected Data (RECORD)” [22].

Ethics

Because this study used only anonymized data, it was not subject to the ethical guidelines set by the Japanese government, and no Ethical Review Committee or informed consent was required [23].

## Results

Prevalence and incidence

**Figure 2:**
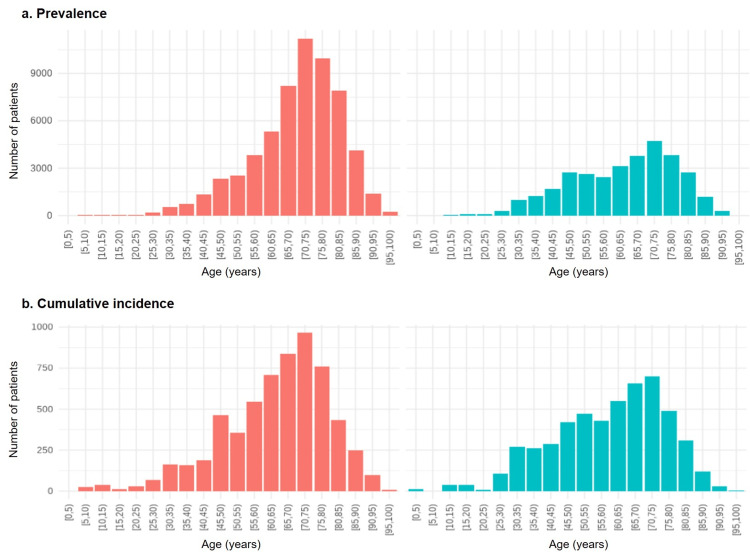
Prevalence and cumulative incidence rates of sarcoidosis in FY2021 according to age group a: Prevalence. b: Incidence Bars: numbers of individuals within five-year age groups; pink bars: females; blue bars: males

Of 25,830 patients with sarcoidosis, 13,115 were included in the calculations of the prevalence and cumulative incidence rates. The demographic estimation based on these data showed a prevalence per 100,000 in FY2021 of 73, and mean ages of 69.7 and 62.6 years for female and male patients, respectively. A total of 1426 new cases were diagnosed in FY2021 (cumulative incidence 9.0 per 100,000 person-years), with mean ages of 63.6 and 59.3 years for female and male patients, respectively. The age and sex distributions of each group are shown in Figure 2.

**Figure 3:**
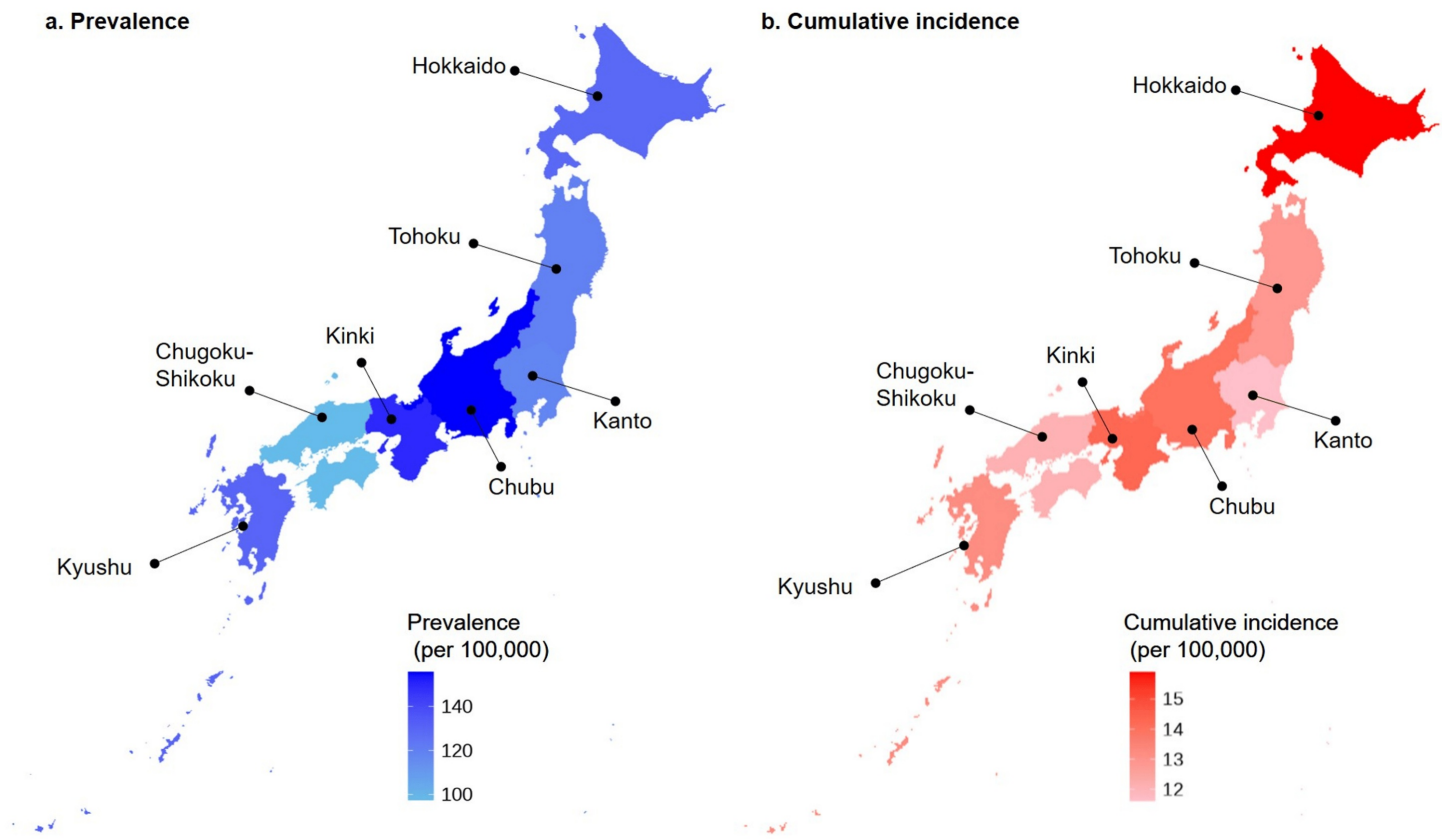
Prevalence and cumulative incidence rates according to region

The prevalence and cumulative incidence rates in each region are shown in Figure 3 and Table 2, respectively.

**Table 2:** Prevalence and cumulative rates according to insurance type and geographical region † Per 100,000 individuals

	National Health Insurance and Advanced Elderly Medical Service System (N=8,255,984)						Health Insurance Association (N=5,327,000)					
	Prevalence			Incidence			Prevalence			Incidence		
	n (%)		rate^†^	n (%)		rate^†^	n (%)		rate^†^	n (%)		rate^†^
Overall	11,263	100%	136.4	1100	100%	13.3	1852	100%	34.8	326	100%	6.1
Hokkaido	162	1.4%	128.8	20	1.8%	15.9	-	-	-	-	-	-
Tohoku	919	8.2%	119.7	99	9.0%	12.9	-	-	-	-	-	-
Kanto	1461	13.0%	117.6	144	13.1%	11.6	-	-	-	-	-	-
Chubu	4922	43.7%	155.7	442	40.2%	14.0	-	-	-	-	-	-
Kinki	2348	20.8%	149.8	224	20.4%	14.3	-	-	-	-	-	-
Chugoku-Shikoku	1075	9.5%	97.4	133	12.1%	12.1	-	-	-	-	-	-
Kyushu	376	3.3%	130.7	38	3.5%	13.2	-	-	-	-	-	-

Patient characteristics

Of the 10,910 cases included in the real-world survey of diagnoses and treatments, 68.8% (n = 7504) were female individuals. The lesion sites were as follows: lung, 32.0% (n = 3493); heart, 20.9% (n = 2283); eye, 14.5% (n = 1577); and skin, 3.4% (n = 369). Other patient characteristics are shown in Table 3. Regarding glycosylated hemoglobin (HbA1c) in FY2021, while data were only available for 2,555 patients (23.4%), of these, 1,274 patients (49.9%) had HbA1c levels ≥5.7%, which is the criterion for prediabetes [24].

**Table 3:** Patient characteristics *Sarcoidosis lesion locations includes duplication N (%), horizontal proportion; n (%), vertical proportion

	Overall		Sarcoidosis lesion locations*							
			Lung		Heart		Eye		Skin	
N (%)	10,910	100%	3493	32.0%	2283	20.9%	1577	14.5%	369	3.4%
Sex, n (%)										
Female	7504	68.8%	2385	68.3%	1479	64.8%	1181	74.9%	293	79.4%
Age, Median (IQR)	77	(67–81)	76	(66–81)	75	(67–80)	77	(68–81)	75	(64–79)
Payment of specific medical expenses										
n (%)	1782	16.3%	804	23.0%	641	28.1%	336	21.3%	89	24.1%

Treatment for sarcoidosis and comorbidities

In FY2021, 25.2% (n = 2547) of patients were prescribed OCS, with a median (interquartile (IQR)) dose of 1645 mg (784-2065) (Table 4).

**Table 4:** OCS prescriptions *Sarcoidosis lesion locations includes duplication OCS, oral corticosteroids

	Overall		Sarcoidosis lesion locations*							
			Lung		Heart		Eye		Skin	
	(N=10,910)		(N=3493)		(N=2283)		(N=1577)		(N=369)	
OCS prescription, n (%)										
0 mg/year	8165	74.8%	2441	69.9%	1229	53.8%	1150	72.9%	256	69.4%
1–1999 mg/year	2000	18.3%	751	21.5%	690	30.2%	311	19.7%	76	20.6%
2000–3999 mg/year	599	5.5%	236	6.8%	293	12.8%	86	5.5%	26	7.0%
4000–7999 mg/year	143	1.3%	64	1.8%	69	3.0%	30	1.9%	11	3.0%
8000– mg/year	3	0.03%	1	0.03%	2	0.1%	0	0.0%	0	0.0%
OCS prescription, mg/year										
Median	1645		1575		1820		1640		1663	
IQR (Q1–Q3)	(784–2065)		(752–2109)		(1400–2497)		(780–2075)		(660–2310)	

Treatments other than OCS are shown in Table 5. The proportion of patients receiving treatment other than OCS, such as immunosuppressants/immunomodulators and biologics, was <3% for all treatments except eye drops.

**Table 5:** Other treatments *Sarcoidosis lesion locations includes duplication ADA, adalimumab; ETN, etanercept; GLM, golimumab; IFX, infliximab; MMF, mycophenolate mofetil; MTX, methotrexate

	Overall		Sarcoidosis lesion locations*							
			Lung		Heart		Eye		Skin	
	(N=10,910)		(N=3493)		(N=2283)		(N=1577)		(N=369)	
Biologics, n (%)										
IFX	7	<0.1%	2	<0.1%	2	<0.1%	2	0.1%		
ADA	46	0.4%	21	0.6%	10	0.4%	17	1.1%	1	0.3%
ETN	11	0.1%	7	0.2%	1	<0.1%	1	<0.1%		
GLM	12	0.1%	9	0.3%	1	<0.1%	2	0.1%		
Immunosuppressants/Immunomodulators, n (%)										
MTX	180	1.6%	87	2.5%	54	2.4%	23	1.5%	7	1.9%
Azathioprine	52	0.5%	16	0.5%	10	0.4%	11	0.7%	1	0.3%
Cyclosporine	41	0.4%	15	0.4%	6	0.3%	8	0.5%	4	1.1%
Tacrolimus	79	0.7%	49	1.4%	14	0.6%	14	0.9%	3	0.8%
Others, n (%)										
MMF	9	<0.1%	4	0.1%	1	<0.1%	1	<0.1%	1	0.3%
Nintedanib	24	0.2%	21	0.6%	1	<0.1%	4	0.3%	1	0.3%
Leflunomide	3	<0.1%	2	<0.1%						
Chloroquine	8	<0.1%	5	0.1%	1	<0.1%	1	<0.1%		
Eye drops, n (%)										
Steroid drugs	3560	33.0%	1138	33.0%	530	23.0%	958	61.0%	151	41.0%
Mydriatics	1738	16.0%	529	15.0%	299	13.0%	445	28.0%	73	20.0%

Table 6 shows the comorbidities reportedly associated with OCS and diseases reportedly comorbid with sarcoidosis. The prevalence of type 2 diabetes and sleep disorders was the highest (50.0% and 36.0%, respectively), followed by rheumatoid arthritis (9.7%) and fractures (8.2%).

**Table 6:** Comorbidities *Sarcoidosis lesion locations includes duplication

	Overall		Sarcoidosis lesion locations*							
			Lung		Heart		Eye		Skin	
	(N=10,910)		(N=3493)		(N=2283)		(N=1577)		(N=369)	
Comorbidities, n (%)										
Type 2 diabetes	5,462	50.0%	1,799	52.0%	1,481	65.0%	741	47.0%	179	49.0%
Sleep disorders	3,962	36.0%	1,267	36.0%	992	43.0%	586	37.0%	127	34.0%
Fractures	899	8.2%	290	8.3%	182	8.0%	135	8.6%	26	7.0%
Spinal fractures	719	6.6%	242	6.9%	148	6.5%	107	6.8%	19	5.1%
Non-spinal fractures	284	2.6%	82	2.3%	60	2.6%	48	3.0%	12	3.3%
Herpes zoster	611	5.6%	206	5.9%	117	5.1%	107	6.8%	31	8.4%
Sleep apnea	274	2.5%	83	2.4%	88	3.9%	34	2.2%	9	2.4%
Tuberculosis	100	0.9%	49	1.4%	20	0.9%	13	0.8%	3	0.8%
Aspergillosis	59	0.5%	37	1.1%	7	0.3%	3	0.2%	1	0.3%
Pulmonary hypertension	88	0.8%	40	1.1%	19	0.8%	11	0.7%	1	0.3%
Autoimmune diseases										
Rheumatoid arthritis	1,058	9.7%	461	13.0%	207	9.1%	154	9.8%	37	10.0%
Sjögren's syndrome	566	5.2%	244	7.0%	63	2.8%	74	4.7%	17	4.6%
Autoimmune thyroiditis	348	3.2%	119	3.4%	73	3.2%	42	2.7%	13	3.5%
Polymyositis	70	0.6%	37	1.1%	18	0.8%	2	0.1%	1	0.3%
Autoimmune hepatitis	58	0.5%	23	0.7%	7	0.3%	7	0.4%	3	0.8%
Vasculitis	49	0.4%	25	0.7%	3	0.1%	6	0.4%		
Multiple sclerosis	35	0.3%	7	0.2%	2	<0.1%	5	0.3%	1	0.3%
Scleroderma	10	<0.1%	3	<0.1%	2	<0.1%	3	0.2%	4	1.1%
Ankylosing spondylitis	5	<0.1%	1	<0.1%	1	<0.1%				

In addition, Table 7 presents the prevalence of comorbidities when defined based on both disease names and prescribed medications. Type 2 diabetes and sleep disorders were observed in 21.6% and 25.1% of patients, respectively.

**Table 7:** Comorbidities (defined by diagnoses and medications) *Sarcoidosis lesion locations includes duplication

	Overall (n=10910)		Sarcoidosis lesion locations*							
			Lung (n=3493)		Heart (n=2283)		Eye (n=1577)		Skin (n=369)	
Comorbidities, n (%)										
Type 2 diabetes	2353	21.6%	711	20.4%	690	30.2%	295	18.7%	85	23.0%
Sleep disorders	2742	25.1%	878	25.1%	674	29.5%	403	25.6%	75	20.3%
Fractures	899	8.2%	290	8.3%	182	8.0%	135	8.6%	26	7.1%
Spinal fractures	719	6.6%	242	6.9%	148	6.5%	107	6.8%	19	5.2%
Non-spinal fractures	284	2.6%	82	2.4%	60	2.6%	48	3.0%	12	3.3%
Herpes zoster	611	5.6%	206	5.9%	117	5.1%	107	6.8%	31	8.4%
Sleep apnea	274	2.5%	83	2.4%	88	3.9%	34	2.2%	9	2.4%
Tuberculosis	18	0.2%	6	0.2%	6	0.3%	2	0.1%		
Aspergillosis	13	0.1%	9	0.3%	2	0.1%	1	0.1%		
Pulmonary hypertension	20	0.2%	9	0.3%	7	0.3%				
Autoimmune diseases										
Rheumatoid arthritis	526	4.8%	253	7.2%	136	6.0%	76	4.8%	19	5.2%
Sjögren's syndrome	394	3.6%	174	5.0%	55	2.4%	56	3.6%	13	3.5%
Autoimmune thyroiditis	148	1.4%	56	1.6%	36	1.6%	16	1.0%	6	1.6%
Polymyositis	50	0.5%	27	0.8%	15	0.7%	1	0.1%	1	0.3%
Autoimmune hepatitis	18	0.2%	9	0.3%	4	0.2%	4	0.3%	1	0.3%
Vasculitis	21	0.2%	12	0.3%	2	0.1%	4	0.3%		
Multiple sclerosis	9	0.1%	1	0.03%						
Scleroderma	4	0.04%	2	0.1%			1	0.1%	1	0.3%
Ankylosing spondylitis	2	0.02%								

A visual overview of the study design, population, and key findings is provided in Appendix B.

## Discussion

This study investigated the prevalence and incidence of sarcoidosis in Japan, along with patient demographics such as sex, age, and treatment patterns, including pharmacological interventions. In FY2021, an estimated 92,101 patients were diagnosed with sarcoidosis in Japan with mean ages of 69.7 years in female individuals and 62.6 years in male individuals. The prevalence was 73 per 100,000 persons and the cumulative incidence was 9.0 per 100,000 person-years. Approximately 25.2% of patients were prescribed OCS, with a median annual dose of 1,645 mg. Prescriptions of nonsteroidal systemic therapies such as immunosuppressants were rare, accounting for <3% of all treatments, excluding eye drops. The most common complications were diabetes mellitus, sleep disorders, and fractures with comorbidity rates of 50%, 36%, and 8.2%, respectively. Similarly, among autoimmune comorbidities, rheumatoid arthritis, Sjögren syndrome, and autoimmune thyroiditis were observed in decreasing order of frequency.

In the present study, the estimated prevalence and cumulative incidence of sarcoidosis were 73 and 9.0 per 100,000 persons, respectively. This is the first epidemiological study in Japan to report these rates based on an analysis of big data. A previous study on patients with sarcoidosis in Japan reported an incidence rate of 1.01 per 100,000 persons [13]. The discrepancy is likely attributable to differences in study populations. Specifically, the present study utilized a claims database that encompassed a broader spectrum of patients with sarcoidosis, including those with milder cases. In contrast, previous studies were limited to patients with severe symptoms, who were certified by the national insurance system and incurred payment of specific medical expenses, as shown in Table 3. When stratified by sex, the cumulative incidence in the present study was 8.5 per 100,000 persons for men and 9.4 per 100,000 persons for women, consistent with previous reports in Japan [13] and also showing a higher prevalence among women. While previous reports suggested higher prevalence and cumulative incidence in the north and lower rates in the south of Japan [25], the big data analysis in the present study revealed no substantial difference between these regions.

In the present study, the distribution of lesion locations was as follows: lung, 32.0% (n = 3,493); heart, 20.9% (n = 2,283); eye, 14.5% (n = 1,577); and skin, 3.4% (n = 369). A previous epidemiological survey conducted in Japan reported that 86% of the patients had pulmonary involvement, 23% had cardiac involvement, 55% had ocular involvement, and 35% had cutaneous involvement. That study further highlighted the significantly higher frequency of eye and skin involvement in women [13]. Similarly, in the present study, the prevalence of eye and skin lesions was also higher in women than in men. However, the proportion of lesions differed from that in earlier studies, except for cardiac involvement. This discordance may be attributed to differences in study methodology. While the previous study utilized a clinical evaluation form for sarcoidosis and diagnosed sarcoidosis based on the clinical presentation and biopsy findings, the present study relied on claims-based data. The claims data included patients categorized as having “unspecified sarcoidosis”, which may have contributed to the differences in the distribution of lesions observed in this study compared to those reported previously.

In FY2021, 25.2% of patients (n = 2,547) were prescribed OCS, with a median (IQR) cumulative dose of 1,645 mg (784-2,065 mg) per year. The daily equivalent dose was approximately 4.5 mg, which is close to the commonly prescribed maintenance dose of 5 mg. However, 6.8% of all patients were prescribed >2,000 mg of OCS annually. Patients with cardiac sarcoidosis were more likely to receive OCS (46%) than those with other types of sarcoidosis, and their median annual dose was higher (1,820 mg). Analysis of claims data in the US reported that 25.5% of patients were administered steroids [26], nearly identical to the proportion observed in the present study. Moreover, a US report demonstrated that a cumulative prednisolone dose >500 mg was significantly associated with an increased risk of hospitalization, whereas the use of methotrexate or azathioprine was associated with a decreased risk of hospitalization [26]. In the current study, the cumulative prednisolone dose was 1,645 mg, and <3.0% of treatments involved immunosuppressive agents other than steroids. Given that no pharmacological therapies other than steroids are currently approved for the treatment of sarcoidosis under the national health insurance system in Japan, the development and implementation of alternative nonsteroidal therapeutic options may be warranted.

In the present study, the comorbidity rates of type 2 diabetes, sleep disorders, spinal fractures, and herpes zoster in patients with sarcoidosis were 52%, 36%, 6.9%, and 5.6%, respectively. The prevalence of type 2 diabetes was higher than that reported in a national epidemiological survey [27]. In the present study, 49.9% of patients had at least prediabetes, defined as an HbA1c level of ≥5.6 in blood tests conducted during health check-ups. This may partly explain the high prevalence rate, as diagnostic labels were assigned specifically for follow-up examinations. Therefore, we combined disease names with corresponding medications to refine the definition of comorbidities. As shown in Table 7, the prevalence of diabetes was 21.6%, which is closer to the rate reported in the previous study [27]. Regarding sleep disorders, a study using the Pittsburgh Sleep Quality Index conducted among 1,871 Japanese adults reported high rates of insomnia and difficulty in maintaining sleep, particularly among elderly individuals. Among female patients in their 70s, the prevalence rates were 26.3% and 24.1%, respectively [28]. For spinal fractures, a database study in Hiroshima reported an incidence rate of 1,558 per 100,000 persons aged ≥65 years, with the number of fractures peaking in the 80s and the incidence rate increasing with age [29]. Regarding herpes zoster, a study in Miyazaki, Japan, reported an incidence rate of 4.79 per 1,000 person-years, with a peak incidence observed in individuals in their 70s [30]. The high comorbidity rates observed in the present study may reflect the increased prevalence of these conditions in older populations and the potential effects of oral corticosteroids prescribed to patients with sarcoidosis. However, as this cross-sectional study was not designed to explore causality, further investigations are warranted to clarify the relationship between corticosteroid use and comorbid conditions.

In the present study, the comorbidity rate of rheumatoid arthritis was 9.7%. The estimated prevalence of rheumatoid arthritis in the general Japanese population is approximately 0.6-1.0% [31]. The high prevalence observed in the present study may be partially attributed to the use of claim-based diagnoses. Despite this limitation, the actual incidence of sarcoidosis and rheumatoid arthritis appears to be higher than that in the general population. A case-control study in the United States on the association between sarcoidosis and autoimmune diseases reported a significant relationship between sarcoidosis and rheumatoid arthritis, with an odds ratio of 1.6 [32]. The pathophysiologies of both diseases primarily involve excessive immune activation triggered by antigen recognition. This activation is characterized by T cell-mediated inflammatory responses involving macrophages and proinflammatory cytokines. Genetic associations with human leukocyte antigen (HLA) have also been suggested [33]. While approximately 70,000 patients in Japan have Sjögren syndrome [31], the comorbidity rate of Sjögren syndrome with sarcoidosis has not been clarified. The present study is the first to report this comorbidity rate (5.2%). Although several case reports have documented the coexistence of sarcoidosis and Sjögren syndrome, differential diagnosis is difficult [34-36]. A Taiwanese study reported a significant association between Sjögren syndrome and sarcoidosis, with an adjusted odds ratio of 11.6 [37]. Both diseases may share immunological features, including elevated CD4+ lymphocyte levels and an association with the HLA-DR3 genotype [38]. The present study found that the comorbidity rate of sarcoidosis with autoimmune thyroiditis was 3.2%. A Spanish study reported a similar comorbidity rate of 2.9% among 348 patients [39]. A Japanese study reported an estimated prevalence of Hashimoto’s thyroiditis in patients with sarcoidosis of 3-11% [40]. In Taiwan, approximately 12% of 1,237 patients with sarcoidosis (predominantly women) had autoimmune thyroid diseases, with an adjusted odds ratio of 1.32, indicating a significant association [37]. Furthermore, a previous review suggested a significantly higher prevalence of autoimmune thyroiditis among female patients with sarcoidosis [41]. The results of the present study are consistent with those reported previously, suggesting a relationship between these diseases. Overall, since the consideration of complications is based solely on claims data, it remains speculative. Further investigation using a well-designed study is necessary to gain more detailed insights.

In Japan, nationwide epidemiological studies on sarcoidosis are limited, especially those that include mild cases or patients under observation without pharmacological treatment. Comprehensive large-scale epidemiological data encompassing the entire spectrum of sarcoidosis are scarce. The results of the present study clarified the total number of patients, their demographics, and the prevalence of sarcoidosis, including mild disease not requiring regular treatment. Data on patient sex, age, and geographic distribution help identify the characteristic epidemiological profile of sarcoidosis, such data informs the development of population-based preventive strategies, optimization of screening programs, and provision of information for clinical guidelines. Moreover, these findings establish a foundation for personalized treatment strategies that consider the relationship between sarcoidosis and its associated comorbidities. Further investigations into the natural history and prognosis of mild cases are anticipated to enhance our understanding of the pathophysiology and clinical management of sarcoidosis.

This study used big data covering approximately 20% of the Japanese population; therefore, the results may be generalizable. In addition, owing to its rarity, surveys, and research on sarcoidosis in actual clinical practice are challenging; however, the big data approach in the present study overcame these issues. Registry data with a more variable range than that provided by claims data would allow the exploration of a wider range of research questions.

Despite these strengths, this study has several limitations. First, because many cases of sarcoidosis listed in the claims data used in this study did not describe the lesions, the findings for each lesion were insufficient. While previous studies have reported that the most common lesion site is in the lung, in this study, the lung was the lesion site in only 32% of patients in the present study. In actual clinical practice for pulmonary sarcoidosis in Japan, physicians often simply enter “sarcoidosis” into the electronic medical record without specifying the organ, and this physician’s behavior may be affecting the results. Moreover, the combination of codes for pulmonary sarcoidosis presented here has not been validated. Second, because the claims data did not include details on severity, the results of this study are a composite of all patients, ranging from mild to severe. Future studies should investigate the characteristics of different disease severities by analyzing databases containing more detailed medical information, such as disease registries. Third, the only data available for this study was claims data, and since there was no gold standard data, validation was not possible. As a result, there remains a possibility that patients who were not actually diagnosed with sarcoidosis may have been misclassified as having the condition, and the prevalence and incidence rates may be subject to overestimation. Finally, the comorbidities identified in this study were based on the codes recorded in the claims data. For example, rheumatoid arthritis was reported in 9.7% of patients; however, this may have been a misclassification reflecting the diagnosis used for testing or prescribing medications rather than confirmed clinical diagnoses. A similar limitation may apply to other autoimmune diseases. Additionally, the prevalence of type 2 diabetes in this study was 50%, which was considerably higher than previous reports. FY21 health check-up data showed prediabetes in 49.9% of the patients. Although we conducted the analysis using a definition based on both disease names and prescribed medications, this approach still has limitations. For instance, it may fail to capture patients who receive lifestyle guidance without pharmacological treatment or those who choose not to initiate medication therapy. Therefore, the diagnosis may have been made through testing and careful follow-up. This would be a strong limitation if the research question was to evaluate patients with type 2 diabetes requiring treatment. To ensure accurate diagnosis, the frequency of diagnosis and combinations with drug treatments should be determined.

## Conclusions

The results of this large-scale epidemiological analysis of claims data provided new insights into sarcoidosis in Japan, including estimated prevalence and cumulative incidence rates of 73 and 9.0 per 100,000 individuals, respectively. Moreover, while approximately one-fourth of patients received oral corticosteroid therapy, other systemic treatments were rarely prescribed. Additionally, the findings elucidated the comorbidity profile of patients with sarcoidosis. These results contribute to our understanding of sarcoidosis in Japan and may help inform clinical practice guidelines and future research.

## Figures and Tables

**Figure 4:**
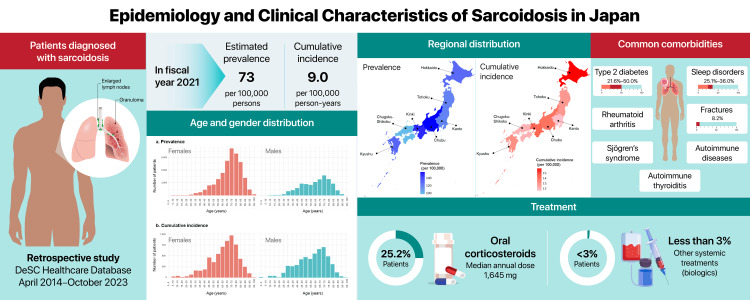
Graphical abstract of the study on sarcoidosis in Japan DeSC, Deidentified Scrubbed Claims Healthcare Database Image credits: Editage (https://www.editage.com)

## Appendices

Appendix A 

**Table 8:** Codes used to define comorbidities ATC, anatomical therapeutic chemical classification; ICD, international classification of diseases and related health problems [15]

Comorbidity	Definition (Diagnosis with treatment)	
	Diagnosis	Treatment
Type 2 diabetes	ICD-10: E11-14	ATC code: A10
Sleep disorders	ICD-10: G470/G472/G478/G479	Generic name: Ramelteon, Zolpidem, Zopiclone, Eszopiclone, Triazolam, Etizolam, Brotizolam, Rilmazafone, Lormetazepam, Nimetazepam, Flunitrazepam, Estazolam, Nitrazepam, Quazepam, Flurazepam, Haloxazolam, Melatonin, Lemborexant, Suvorexant
Fractures	Disease name: "Fracture"	
Herpes zoster	Disease name: "Herpes zoster"	
Sleep apnea	Disease name: "Sleep apnea"	
Tuberculosis	Disease name: "Tuberculosis"	ATC code: J04A
Aspergillosis	Disease name: "Aspergillosis"	ATC code: J02A
Pulmonary hypertension	Disease name: "Pulmonary hypertension"	ATC code: C06B
Rheumatoid arthritis	Disease name: "Rheumatoid arthritis"	ATC code: H02A2/L04B/M01 Generic name: Tofacitinib, Baricitinib, Peficitinib, Upadacitinib, Filgotinib
Sjögren's syndrome	Disease name: "Sjögren's syndrome"	ATC code: H02A2, S01K Pilocarpine (oral, ophthalmic), Cevimeline (oral), Saliva substitute (Dipotassium phosphate・Potassium chloride・Calcium chloride hydrate・Sodium chloride・Magnesium chloride)
Autoimmune thyroiditis	Disease name: "Autoimmune thyroiditis"	ATC code: H03A
Polymyositis	Disease name: "Polymyositis"	ATC code: H02A2 Generic name: Azathioprine, Cyclosporine, Tacrolimus, Cyclophosphamide, Mycophenolate mofetil, Methotrexate
Autoimmune hepatitis	Disease name: "Autoimmune hepatitis"	ATC code: H02A2 Generic name: Azathioprine
Vasculitis	Disease name: "Vasculitis"	ATC code: H02A2 Generic name: Methotrexate, Cyclophosphamide, Rituximab, Tocilizumab, Azathioprine, Avacopan
Multiple sclerosis	Disease name: "Multiple sclerosis"	ATC code: N07A0 Generic name: Carbamazepine
Scleroderma	Disease name: "Scleroderma"	ATC code: H02A2 Generic name: Rituximab
Ankylosing spondylitis	Disease name: "Ankylosing spondylitis"	Generic name: Sulfasalazine, Infliximab, Adalimumab, Secukinumab, Ixekizumab, Brodalumab, Bimekizumab, Upadacitinib

Appendix B 
